# Symptoms and markers of symptom severity in asthma—content validity of the asthma symptom diary

**DOI:** 10.1186/s12955-015-0217-5

**Published:** 2015-02-13

**Authors:** Gary Globe, Mona Martin, Michael Schatz, Ingela Wiklund, Joseph Lin, Robyn von Maltzahn, Maria S Mattera

**Affiliations:** Global Health Economics, Amgen Inc., One Amgen Center Drive MS 28-3-A, Thousand Oaks, CA 91320 USA; Health Research Associates, Inc., 6505 216th St SW, Mountlake Terrace, WA 98043 USA; Department of Allergy, Kaiser Permanente Medical Center, 4647 Zion Ave, San Diego, CA 92120 USA; Evidera, Metro Building, 6th Floor, 1 Butterwick, London, W6 8DL UK; Evidera, 7101 Wisconsin Ave, Suite 600, Bethesda, MD 20814 USA; Currently with Gilead Sciences, Inc., Foster City, CA USA

**Keywords:** Asthma, Symptoms, Patient-reported outcome, Instrument development, Qualitative

## Abstract

**Background and objectives:**

The American Thoracic Society/European Respiratory Society (ATS/ERS) Task Force acknowledged the multi-faceted nature of asthma in its recent definition of asthma control as a summary term capturing symptoms, reliever use, frequency/severity of exacerbations, lung function, and future risk and the Global Initiative for Asthma (GINA) defines the clinical manifestations (well established markers of asthma severity) of asthma to include symptoms, sleep disturbances, limitations of daily activity, impairment of lung function, and use of rescue medications. The objectives of this qualitative work were to identify symptoms and markers of symptom severity relevant to patients with moderate to severe asthma and to evaluate the content validity of the asthma symptom diary (ASD).

**Methods:**

A qualitative interview study was conducted using a purposive sample of symptomatic adult and adolescent (≥12 years) subjects with asthma. Concept elicitation (CE) interviews (n = 50) were conducted to identify core asthma symptoms and symptom-related clinical markers, followed by cognitive interviews (n = 24) to ensure patient comprehension of the items, instructions and response options. CE interviews were coded using ATLAS.ti for content analysis.

**Results:**

The study sample had a diverse range of symptom severity, level of symptom control, sociodemographic and socioeconomic status. The most frequently reported symptoms in adults were chest tightness (n = 33/34; 97.1%), wheezing (n = 31; 91.2%), coughing (n = 30; 88.2%), and shortness of breath (n = 25; 73.5%); in adolescents they were wheezing (n = 14/16; 87.5%), coughing (n = 13; 81.3%), and chest tightness (n = 11; 68.8%). Adults identified chest tightness followed by shortness of breath as their most severe symptoms; while adolescents reported coughing and chest tightness as their most severe symptoms. Sleep awakenings and limitations in day-to-day activities were frequent symptom-related clinical markers. Day-to-day variability and differences between daytime and nighttime symptom experiences reported by subjects resulted in the need for the ASD to be administered twice daily. Cognitive interviews indicated that subjects found the revised ASD items clear and easy to understand.

**Conclusions:**

This study supports the content validity of the revised ASD, showing it to be consistent with patient experiences and ready for further psychometric testing.

## Background

Asthma is a serious, highly prevalent disease which affects all age groups, with an estimated 1 in 13 people affected in the United States (US) [1]. Globally, 300 million people suffer from asthma with 250,000 deaths attributable to asthma each year [2] and a prevalence of 8.2% which has increased annually between 2001 and 2009 at a rate of 1.2% [3]. With around 12.8 million people (8.7 million adults and 4 million children (aged 0–17)) having an attack within the last year, resulting in 10.5 million missed school days and 14.2 million missed work days in 2008 [3], asthma has a significant societal impact and is a serious public health problem [4].

Airflow limitation measured by forced expiratory volume in one second (FEV_1_), has been, and still is, an important endpoint used in asthma clinical trials [5,6]. However, there is growing recognition that traditional clinical outcomes, such as lung function, are inadequate indicators of how asthma patients function and feel [7]. The multifactorial and complex nature of asthma control [8] is supported by evidence indicating poor correlations between lung function and symptoms and between lung function and ß-agonist usage [9,10].

The American Thoracic Society/European Respiratory Society (ATS/ERS) Task Force acknowledged the multi-faceted nature of asthma in its recent definition of asthma control as a summary term capturing symptoms, reliever use, frequency/severity of exacerbations, lung function, and future risk [5] and the Global Initiative for Asthma (GINA) defines the clinical manifestations (well established markers of symptom severity) of asthma to include symptoms, sleep disturbances, limitations of daily activity, impairment of lung function, and use of rescue medications [4].

Clinicians tend to underestimate asthma symptom severity, making the use of a patient-completed daily diary for the assessment of asthma symptoms and markers of symptom severity in clinical trials essential [11-13]. As identified by the National Institutes of Health (NIH) Asthma Outcomes Workshop [14] and further confirmed by an internally conducted focused literature review, there is no current asthma diary that can be recommended for use due to the lack of published validation data or appropriate recall period.

The Asthma Control Questionnaire (ACQ) is widely regarded as a useful measure that includes some core asthma symptoms and symptom-related clinical markers of asthma. While the ACQ is designated as a core measure in NIH-initiated clinical research [8], the seven-day recall period represents a potential shortcoming in the context of FDA recommendations for symptom measures in clinical trials [15]. Given the variable nature of asthma symptoms, a measure with a shorter recall (i.e., daily), is preferable. Also, while self-reported use of rescue medication is important to capture, its incorporation into a symptom scale may introduce some limitation in symptom scale interpretation (i.e., to what extent do changes in the symptom score simply reflect changes in rescue medication use rather than reflecting a true change in symptoms).

Prior to the Food and Drug Administration (FDA) Guidance for PROs, Amgen had developed an electronic patient-reported outcome (ePRO) diary to assess asthma symptoms and rescue medication use on a daily basis. Following the publication of the FDA Guidance, it was recognized that the measure required additional study to evaluate and document its content validity [16,17]. Following the recommendations in the FDA guidance, qualitative patient interviews were conducted to elicit relevant concepts. Subsequently, cognitive interviews were conducted to assess patient comprehension of the concepts presented by the diary items. As the measure under development was intended to be a daily diary, the team, comprising clinical and PRO measurement experts, decided that capturing the broader range of asthma impacts was not feasible. However, as reflected in the literature [18] the effect on sleep and activities are key markers of symptom severity. Therefore a decision was made that, if supported by patient qualitative research, these two items would be retained (at least in the qualitative phase of study) as markers of symptom severity.

This work aimed to evaluate the content validity of the asthma symptom diary (ASD) for use as an efficacy endpoint in asthma clinical trials to support labelling claims. This article describes the qualitative research phase documenting content validity in adult and adolescent asthma patients, and the resulting revisions. The evaluation of the cross-sectional measurement properties of the ASD, conducted subsequent to the qualitative research, will be reported elsewhere [19].

## Methods

Concept elicitation interviews (n = 50) and cognitive interviews (n = 24) were conducted in adults and adolescent (≥12 years) who had a clinical diagnosis of persistent asthma. Four sites, asthma care clinical practices, in different regions of the US were used to recruit patients for the interviews. Institutional Review Board (Essex IRB Inc.) approval was obtained prior to study initiation.

### Subjects

Subjects were screened for eligibility, enrolled, and scheduled for one of the two types of qualitative interviews. Included subjects had an asthma diagnosis for at least one year, were on asthma treatment maintenance medication regimen, and were either non-smokers or ex-smokers (<10 pack years and stopped ≥1 year ago). Subjects were not included if they had any major health problems interfering with asthma or were not able to give informed consent. Additionally, the study was designed to ensure that patient clinical and demographic characteristics were similar to those to be included in the planned clinical trial program.

### Study measures

As part of the enrollment process, patients completed a self-administered version of the Asthma Control Questionnaire (ACQ-7) and the Asthma Quality of Life Questionnaire (AQLQ).

The ACQ-7 [20] is a seven-item instrument assessing asthma control during the past week. The ACQ includes six patient-reported items about nighttime awakening, symptom severity upon awakening, activity limitation due to asthma, shortness of breath due to asthma, wheezing, and use of a short-acting bronchodilator. The final item is the forced expiratory volume in one second (FEV_1_) % predicted completed by clinicians following spirometry. Items are scored 0 (good control) to 6 (poor control), and the total ACQ score is the mean of the seven items.

AQLQ12+ (12 years and older) [21] contains 32 items comprising four domains: Activity Limitation, Asthma Symptoms, Emotional Function, and Environmental Stimuli. Items are scored 1 (severe impairment) to 7 (no impairment), with domain scores calculated as the mean of the items within each domain; the overall AQLQ12+ score is the mean of all 32 items.

### Clinical and sociodemographic forms

Clinicians provided additional descriptive information from the patient records and subjects completed a sociodemographic form that included patients’ date of birth, gender, race/ethnicity, years of education, employment status, and income level.

### Concept elicitation interviews

The first phase of the qualitative work consisted of individual concept elicitation interviews with 34 adults and 16 adolescent subjects having a diagnosis of asthma. Each face-to-face individual interview was conducted privately and lasted approximately 60 minutes. The semi-structured interview guide asked patients to describe their experience with asthma symptoms and symptom related impacts during a typical day. Rating exercises for symptom bothersomeness and degree of difficulty patients experienced in coping with their asthma were conducted at the end of each interview. Interview results were used to identify the most relevant concepts to the patient experience, and the predominant terminology used by patients in identifying their asthma symptoms and impacts.

Following the concept elicitation interviews an item generation meeting was convened to review the qualitative data and revise and/or create new items to the existing items in the earlier diary. This meeting was attended by clinical experts in the treatment of asthma, PRO development experts, and members of the sponsor’s team.

### Cognitive interviews

The cognitive interviews were subsequently conducted in 15 adults and 9 adolescents to assess the patients’ comprehension of the items, the clarity of response options, and the overall feasibility of the measure (format, instructions, appropriate attribution, and language) in the revised version of the ASD. The cognitive interviews were conducted in an iterative process using three waves with evaluation and revision of the diary items following each wave of interviews.

### Analysis

All interviews were digitally recorded and then transcribed. ATLAS.ti software was used to organize the codes being assigned to the transcript data. Descriptive statistics from screening and enrollment data were used to further describe the study population.

A concept saturation table was used to identify the point at which no new concepts were being identified in the data. The transcripts were ordered chronologically based on interview date, and grouped into quartiles. The codes of each subsequent transcript group were compared with those from the prior group. If new codes appeared in subsequent group, it would suggest that saturation had not been achieved.

Translatability (cross-cultural applicability) and reading comprehension level (lexibility) assessments were conducted. All alterations made to the ASD items throughout the process were recorded in an item tracking matrix.

## Results

### Sociodemographic and clinical characteristics

Sociodemographic and clinical characteristics are outlined in Tables 1 and 2. The sample reflects diversity in terms of disease severity, gender, age (both adults and adolescents), education, ethnicity, and socioeconomic status, and was aimed to reflect the general asthma population. Disease control, as defined by ACQ score, was broad (ranging from 0.14-3.8) in order to ensure that the ASD items would be suitable for use across patients with the full spectrum of controlled and uncontrolled asthma.

**Table 1:** **Demographic characteristics of study subjects**

	**Concept elicitation interviews**		**Cognitive interviews**	
	**Adult N = 34 (100%)**	**Adolescent N = 16 (100%)**	**Adult N = 15 (100%)**	**Adolescent N = 9 (100%)**
**Age (years):**				
Mean (SD)	38.9 (13.0)	15.2 (1.6)	30.7 (9.7)	14.1 (2.2)
**Gender:**				
Male	13 (38.2%)	9 (56.3%)	2 (13.3%)	7 (77.8%)
Female	21 (61.8%)	7 (43.8%)	13 (86.7%)	2 (22.2%)
**Marital status:**				
Married or living as married	14 (41.2%)	---	5 (33.3%)	---
Widowed	1 (2.9%)	---	1 (6.7%)	---
Divorced	5 (14.7%)	---	2 (13.3%)	---
Never married	14 (41.2%)	16 (100.0%)	7 (46.7%)	9 (100.0%)
Education*:				
Elementary school	---	6 (37.5%)	2 (13.3%)	6 (66.7%)
High school	3 (8.8%)	10 (62.5%)	1 (6.7%)	3 (33.3%)
College	29 (85.3%)	---	10 (66.7%)	---
Graduate or professional school	2 (5.9%)	---	2 (13.3%)	---
**Employment outside home:**				
Full-time	18 (52.9%)	---	7 (46.7%)	---
Part-time	8 (23.5%)	2 (12.5%)	6 (40.0%)	---
Retired	2 (5.9%)	---	---	---
Not employed	6 (17.6%)	14 (87.5%)	2 (13.3%)	9 (100.0%)
**Ethnic group:**				
White (Non-Hispanic)	19 (55.9%)	8 (50.0%)	12 (80.0%)	5 (55.6%)
Black/African American	12 (35.3%)	4 (25.0%)	1 (6.7%)	---
Asian/Pacific Islander	1 (2.9%)	2 (12.5%)	2 (13.3%)	---
Hispanic/Latino	2 (5.9%)	2 (12.5%)	---	4 (44.4%)

*Highest level for adults; current level for adolescents.

**Table 2:** **Clinical characteristics of study subjects**

	**Concept elicitation interviews**		**Cognitive interviews**	
**Parameter**	**Adult N = 34 (100%)**	**Adolescent N = 16 (100%)**	**Adult N = 15 (100%)**	**Adolescent N = 9 (100%)**
**Atopic status**				
Allergic	27 (79.4%)	16 (100.0%)	10 (66.7%)	9 (100.0%)
Non-allergic	5 (14.7%)	---	4 (26.7%)	--
Missing	2 (5.9%)	---	1 (6.7%)	--
**FEV**_**1**_ **predicted:**				
>95%	3 (8.8%)	8 (50.0%)	4 (26.7%)	2 (22.2%)
95%–90%	3 (8.8%)	2 (12.5%)	1 (6.7%)	2 (22.2%)
89%–80%	10 (29.4%)	5 (31.3%)	2 (13.3%)	3 (33.3%)
79%–70%	9 (26.5%)	---	5 (33.3%)	2 (22.2%)
69%–60%	4 (11.8%)	1 (6.3%)	2 (13.3%)	---
59%–50%	3 (8.8%)	---	1 (6.7%)	---
<50%	2 (5.9%)	---	---	---
**ACQ scores**	**Adult N = 34 (100%)**	**Adolescent N = 16 (100%)**	**Adult N = 15 (100%)**	**Adolescent N = 9 (100%)**
**ACQ score categories:**				
ACQ score ≥3 (severe group)	7 (20.6%)	5 (31.3%)	3 (20.0%)	1 (11.1%)
ACQ score ≥1.5 to < 3 (moderate group)	23 (67.6%)	10 (62.5%)	9 (60.0%)	6 (66.7%)
ACQ score ≥0.5 to <1.5 to (mild group)	4 (11.8%)	1 (6.3%)	3 (20.0%)	2 (22.2%)
**ACQ mean score:**				
Mean (SD)	2.1 (0.74)	1.7 (0.63)	2.1 (0.80)	2.3 (0.90%)
**AQLQ: Overall score**	**Adult N = 34 (100%)**	**Adolescent N = 16 (100%)**	**Adult N = 15 (100%)**	**Adolescent N = 9 (100%)**
Mean (SD)	4.8 (1.2)	5.0 (0.8)	4.8 (1.0)	5.0 (1.1)
Median	4.9	5.2	4.8	5.3
Range	2-7	4-7	3-7	3-6

### Concept elicitation interviews

#### Asthma symptoms

The most frequently reported symptoms in the adult population were chest tightness (n = 33/34; 97.1% of patients reporting this symptom), wheezing (n = 31; 91.2%), coughing (n = 30; 88.2%), and shortness of breath (n = 25; 73.5%). In adolescent subjects, wheezing (n = 14/16; 87.5%), coughing (n = 13; 81.3%), chest tightness (n = 11; 68.8%), and fatigue (n = 9; 56.3%) were the most frequently reported symptoms. The most difficult symptoms of asthma were shortness of breath (mean rank = 7.2), followed by chest tightness, coughing, and wheezing (6.7, 6.3, and 6.0, respectively). The severity scores rate the severity of each symptom on a 10-point NRS and indicate that chest tightness (mean score = 7.6), shortness of breath, coughing, and wheezing (mean scores of 7.3, 7.0, and 7.0, respectively) were the most intensely experienced symptoms by adults and also reported to be among the most important symptoms to alleviate. The adolescent group’s most difficult reported symptom was fatigue (mean rank = 7.4), followed by chest tightness, wheezing, and coughing (6.6, 6.5, and 6.3, respectively). For adolescents, the most severe symptoms were coughing and chest tightness (mean rating = 6.9 for both), followed by fatigue, shortness of breath, and wheezing (6.7, 6.6, and 6.6, respectively).

#### Markers of symptom severity in asthma

The two most frequently reported symptom-related clinical manifestations (markers of symptom severity as endorsed by GINA), were physical activities (adult, n = 25 spontaneous mentions; adolescent, n = 12 spontaneous mentions) and sleep disruption (adult, n = 14 spontaneous mentions; adolescent, n = 8 spontaneous mentions).

#### Variations – age and timing

While activity limitation for adolescents tended to focus more on physical activities tied to school, and adults tended to speak more about restrictions in activities commonly engaged in as a part of their daily life, both groups tended to use the same type of descriptors for the activities and the degrees of restriction they experienced. For example, adolescent subjects used different reference for their activities (i.e., physical education class versus the adults’ expression of exercising at the gym). Even though adult and adolescent subjects referred to different forms of activities, the focus in both age groups was on the same aspect of restriction and impairment.

During the concept elicitation interviews, subjects were asked to describe whether they experienced each symptom more often or with more severity during the day or at night. Table 3 shows a breakdown of subject expressions regarding whether they experienced specific symptoms more frequently and/or severely during the day or night. It can be seen that subjects spoke about experiencing certain symptoms, such as breathing difficulties, more often and more severely during the day and some as more often and more severely at night (such as chest discomfort or wheezing).

**Table 3:** Number of day and nighttime patient language symptom expressions of frequency and severity*

**Symptoms**	**Most frequent**				**Most severe**			
	**Day**		**Night**		**Day**		**Night**	
	**Adults**	**Adolescents**	**Adults**	**Adolescents**	**Adults**	**Adolescents**	**Adults**	**Adolescents**
Breathing difficulties	18	8	7	2	13	6	6	3
Wheezing	9	4	13	4	7	1	9	8
Coughing	6	3	12	6	7	1	7	6
Chest discomfort	13	3	8	6	8	1	9	5
Tiredness	4	2	1	0	5	2	0	0
Dizziness and lightheadedness	2	1	0	0	0	1	0	1
Allergic symptoms	0	0	1	0	1	0	0	0
Throat closing	0	0	1	1	0	0	0	0

*Table reflects the number of participants who indicated whether symptom frequency and symptom severity was worse during the day or night.

Saturation of concepts was achieved in both adult and adolescent study population subgroups by the end of the third transcript group, with no new concepts elicited from the last interviews in the study sample.

After extensive review of the data and discussion at the item generation meeting, the following points and modifications were agreed upon:

#### Decisions made based on the concept elicitation and supported by the literature

- The four asthma symptoms that appeared to be the most relevant to patients included shortness of breath, chest tightness, coughing, and wheezing. These were selected for inclusion in the diary as specific symptom items. Night awakenings and activity limitations (considered symptom-related clinical manifestations by GINA, also referred to as markers of symptom severity) were the two most frequently reported impacts among both adolescents and adults and these were retained as well. Based upon the qualitative evidence as well as clinical and PRO expert opinion, no new items needed to be added to the ASD.
- Severity was reported by patients as the most logical aspect to be asked about when they reported their symptoms. Questions stems were changed to specifically ask patients to report on the severity of their symptoms rather than simply rate their symptoms and rely on the response option to sufficiently carry the message of what they were being asked to think about.
- It was agreed that a 5-point ordinal severity scale (no symptom to very severe) should replace the original 4-point scale for all items in the ASD to better capture the range of symptom severity.
- The qualitative evidence indicated that symptom severity differed between night and day. Therefore it was determined that the same symptom items should be added to the morning diary.

#### Decisions based on cognitive interviews

The results of initial two waves of cognitive interviews highlighted the need to make several relatively minor modifications. For example, items with duration as a response, symbols used to indicate more than or less than were replaced with full wording to improve clarity.

At the conclusion of wave 3, patients reported finding the measure understandable, relevant to their experience, and easy to complete. The response options were endorsed as understandable and matching the item stem by the end of the third wave of interviews.

The final revised ASD is an 11-item measure that is comprised of 6 items from the morning diary and 5 items from the evening diary. The morning diary items include questions about the severity of wheezing, shortness of breath, cough, chest tightness, and the presence and duration of nighttime awakenings. The evening diary items include questions about the severity of wheezing, shortness of breath, cough, chest tightness, and activity limitation.

In addition to the 11 ASD items (Table 4; 9 symptoms and 2 markers of symptom severity) described above, there are an additional 10 items that are not a scored part of the ASD but provide important clinical information on the patient status alongside symptom severity. These additional 10 items (5 morning and 5 evening) ask about the use of asthma rescue medication. The rescue medication-related questions cover the frequency of rescue inhaler or nebulizer use. The rescue medication questions are not part of the ASD score or 7-day average ASD symptom severity score. The rescue medication questions are summarized in a separate daily and weekly rescue medication score.

**Table 4:** **ASD Items**

	**ASD items**	**5 Item response range**
	**Morning (AM)**	
AM 1	Wheezing	None – Very severe
AM 2	Shortness of breath	None – Very severe
AM 3	Cough	None – Very severe
AM 4	Chest Tightness	None – Very severe
AM 5	Nighttime awakenings	Zero – Unable to sleep
AM 6	Length of time awake	Slept through the night – Awake more than 3 hours
	**Evening (PM)**	
PM 6	Wheezing	None – Very severe
PM 7	Shortness of breath	None – Very severe
PM 8	Cough	None – Very severe
PM 9	Chest tightness	None – Very severe
PM 10	Activity limitations	Not at all – Extremely

ASD = Asthma symptom diary.

## Discussion

This qualitative research identifies a set of symptoms and markers of symptom severity that are relevant to the experience of patients who have asthma. The most relevant symptoms identified in both the adult and adolescent populations included chest tightness, wheezing, coughing, and shortness of breath. These symptoms, identified in the qualitative research, are consistent with the core asthma symptoms identified in the literature [5,18]. Among impacts that are considered to be markers of symptom severity, limitations in physical activities and sleep disruption were identified in this qualitative research study as being most difficult for patients to cope with. The decision to include these two markers of symptom severity in the ASD was supported by both the qualitative research (patient spontaneous self-report) as well as the literature [8,18], which indicates that physical activity and sleep problems feature prominently in asthma. Although activity limitations and sleep disturbances might also be considered as assessing impact, the study team, influenced by the weight of the evidence in the literature, theorized that, in the case of asthma, symptom severity may be expressed in terms of their impact. The study team conceptualized that one or more markers of symptom severity could be so interrelated to symptoms that they might scale together with symptoms. A well-established indicator of asthma symptom severity is interference with sleep and activity limitation. Although these could be considered impacts rather than symptoms per se, they were included as well-established markers of symptom severity, and were endorsed during the qualitative phase and confirmed during the quantitative phase.

Fatigue was another symptom raised in the interviews but it was decided by the team of clinician and PRO experts not to include it as it was posited that it may be a symptom that is not be solely attributable to asthma. Because asthma symptoms frequently vary within a 24-hour period, the team decided to include symptoms in both the morning and evening ASD to accurately capture this variation. During the development process, the ASD item wording was revised to ensure that the items were understandable to patients with lower reading levels, as well as to adolescents. Consideration was also given to selecting language usage that was easily translatable.

The ASD is a newly developed PRO intended for use in clinical trials to support labeling claims. The ASD was developed in line with FDA guidance in terms of content validity in a population similar to the clinical trial population, and includes evidences to support concept relevance, saturation of concept, and appropriate understanding of the concepts presented on the part of the patient. The ASD content, structure, and relevance to intended measurement strategy for assessing the severity of asthma symptoms is supported by input from qualitative interviews with patients, consultation and revision by clinical experts, by asthma literature, and by PRO experts.

It is important to note the considerable overlap between the markers of symptom severity of asthma as defined by GINA (symptoms, sleep disturbances, limitations of daily activity, impairment of lung function, and use of rescue medications) and those identified by participants in this qualitative research. As there are established clinical measures for assessing lung function, this aspect was not explored for inclusion in the ASD; however, all other aspects of asthma, as contemplated by GINA, emerged in the qualitative subject interviews and, in addition to the clinical endorsement of these symptoms throughout the development process, add support to the content validity of the ASD.

Traditionally the assessment of asthma has included clinical factors such as lung function and exacerbation rates. Patient-reported and clinical composite measures have also been used to assess the patient experience although none presently meet the rigorous standards reflected by current FDA guidance [15]. This together with the evidence that lung function [7] and clinician reported severity [11-13] do not necessarily reflect a patient’s experience of asthma and highlights the need for a more accurate assessment of asthma outcomes. Moving forward, in accordance with recent consensus recommendations [4,5,22], if studies are to accurately assess treatment options, then they need to accurately assess multiple asthma outcomes. Symptoms are one of the key elements that require attention and this need is reiterated in the review by Krishnan et al. [14] which did not find a single measure suitable for recommendation as a “core” symptom measure. Thus, the development of a patient-reported symptom diary, with documented content validity, serves an important need; to obtain the patients’ perspective on treatment outcomes that are not presently accurately captured in other endpoint assessments.

Qualitative research confirmed the overall content of the original clinician developed ASD. Expert clinicians reviewing the qualitative results agreed that there were no additional asthma symptoms that needed to be added to the diary. Refinements to item language and response items were based upon the results of the qualitative research with patients in conjunction with significant input from clinicians and PRO experts. The similar manner in which both adults and adolescents reported and understood their experiences suggested that one single measure for use in both groups was found to be appropriate. As there was a clear distinction between day and night symptoms for both groups, it was determined that the diary should be administered twice in a 24-hour period—once upon awakening in the morning and once prior to retiring to bed in the evening.

The ASD consists of a single domain assessed twice-daily via an electronic device. The ASD has documented content validity evidenced by qualitative research with asthma patients. This qualitative research study was performed in line with the recommendations in the FDA PRO Guidance. Evaluation of cross-sectional measurement properties has been completed, further confirming unidimensionality (by factor analysis) and content validity (by Rasch analysis), and will be reported in detail in a separate publication.

## Conclusions

The development of the ASD was in line with recommendations in the FDA PRO Guidance document, with key input from patients, clinicians and the literature. Revisions were made during the development process based on patient responses, clinical advice and expertise in the development of PROs. Patients included in this research reflected a diverse asthma population in terms of disease control, gender, age (both adults and adolescents), education, ethnicity, and socioeconomic status mirroring the general asthma population as well as the intended trial population of adults and adolescents with persistent asthma. In the concept elicitation interviews concept saturation and content validity was confirmed. The outcome of this study suggests that the ASD has demonstrated content validity. The resulting 11-item morning and 10-item evening diary assessing asthma symptoms and markers of symptom severity, instructions, and response options are well understood and relevant as verified by patients with asthma. This study supports the content validity of the revised ASD, showing it to be consistent with patient experiences and ready for further psychometric testing. The results of the quantitative assessment of the psychometric properties of the revised ASD are reported elsewhere.

## Abbreviations

ACQ: Asthma Control Questionnaire
AQLQ: Asthma Quality of Life Questionnaire
ASD: Asthma symptom diary
ATS: American Thoracic Society
eDiary: Electronic diary
ePRO: Electronic patient-reported outcome
ERS: European Respiratory Society
FDA: Food and Drug Administration
FEV_1_: Forced expiratory volume in one second
GINA: Global Initiative for Asthma
NIH: National Institutes of Health
NRS: Numerical rating scale
US: United States
